# Impact of Antidepressants on Weight Gain: Underlying Mechanisms and Mitigation Strategies

**Published:** 2025-05-05

**Authors:** Michael Mouawad, Leena Nabipur, Devendra K. Agrawal

**Affiliations:** Department of Translational Research, College of Osteopathic Medicine of the Pacific, Western University of Health Sciences, Pomona, California 91766 USA

**Keywords:** Antidepressant, Bupropion, Dopaminergic pathway, GLP-1 receptor agonist, Metabolic effect, Monoamine oxidase inhibitor, Norepinephrine-dopamine reuptake inhibitor, Pharmacogenomics, Selective serotonin reuptake inhibitor (SSRI), Serotonergic pathway, Tetracyclic antidepressant, Tricyclic antidepressant, Weight gain

## Abstract

Antidepressants are widely prescribed for major depressive disorder and anxiety, yet their long-term use is associated with weight gain, affecting up to 55–65% of patients. This adverse effect contributes to treatment discontinuation, relapse, and worsened metabolic health outcomes, including increased risk for obesity and type 2 diabetes. This artic le presents a critical evaluation of the published reports on the mechanisms underlying antidepressant-induced weight gain, comparative effects across drug classes, and mitigation strategies. Weight gain varies significantly by antidepressant class. Tricyclic antidepressants, monoamine oxidase inhibitors, and a tetracyclic antidepressant, mirtazapine, are associated with the most substantial weight increases, while selective serotonin reuptake inhibitors typically induce weight gain after prolonged use. Mechanisms involve serotonergic and dopaminergic signaling, receptor desensitization, insulin resistance, and altered leptin and ghrelin levels. Genetic factors, including CYP2C19 metabolizer status, and lifestyle factors such as baseline body mass index and diet, further influence risk. Bupropion, a norepinephrine-dopamine reuptake inhibitor, is the only commonly prescribed antidepressant consistently associated with weight loss or neutrality. Mitigation strategies include switching medications, adding agents like metformin or GLP-1 receptor agonists, and incorporating behavioral interventions. Antidepressant-induced weight gain is a multifactorial issue requiring individualized management. Understanding pharmacologic mechanisms and patient-specific risk factors is essential for optimizing treatment efficacy while minimizing metabolic burden.

## 1. Introduction

Major depressive disorder (MDD) is a major global public health concern, affecting over 264 million individuals worldwide [1]. Currently, antidepressants are the most prescribed medications for psychiatric disorders and are the first-line pharmacologic treatment for MDD and anxiety disorders [2]. Additionally, they are used for various in-label and off-label indications, including insomnia, pain, migraines, and eating disorders [3–7]. While antidepressants have shown comparable efficacy across different classes, studies have shown a significant increase in discontinuation rates compared to placebo groups and one of the most common and concerning adverse effects of long-term antidepressant use is weight gain, which could affect as much as 55–65% of those on antidepressant therapy [8–10].

There are multiple classes of antidepressants, each with different mechanisms of action. Selective serotonin reuptake inhibitors (SSRIs) and serotonin-norepinephrine reuptake inhibitors (SNRIs) are typically considered first-line treatments for MDD. However, the selection of an antidepressant should be individualized based on prior treatment response, adverse effect profiles, and patient preference [8,11–12]. SSRIs, such as fluoxetine, sertraline, and citalopram, act by inhibiting the serotonin transporter (SERT), leading to increased serotonin levels in the synaptic cleft and enhanced serotonergic neurotransmission [13,14]. SNRIs, including venlafaxine and duloxetine, work similarly but also inhibit the norepinephrine transporter (NET), which increases both serotonin and norepinephrine levels [15,16].

Other classes of antidepressants include tricyclic antidepressants (TCAs), monoamine oxidase inhibitors (MAOIs), and atypical antidepressants. TCAs, such as amitriptyline and nortriptyline, inhibit both SERT and NET but also interact with histaminic, cholinergic, and alpha-adrenergic receptors, expanding their side effect profile [13,17]. Because of this, TCAs are typically reserved for treatment-resistant depression [18]. MAOIs, such as phenelzine, prevent the breakdown of serotonin, norepinephrine, and dopamine via inhibition of monoamine oxidase enzymes [19,20]. While effective, MAOIs, like TCAs, are generally used as a last resort due to dietary restrictions and potentially severe drug interactions [18]. Atypical antidepressants, such as bupropion and mirtazapine, have unique mechanisms. Bupropion is a norepinephrine-dopamine reuptake inhibitor (NDRI) with less effect on serotonin compared to other antidepressants. It is often prescribed to those experiencing sexual dysfunction with SSRIs or for smoking cessation [21,22]. Mirtazapine, a noradrenergic and specific serotonergic antidepressant (NaSSA), increases neurotransmission through presynaptic alpha-2-adrenergic receptor antagonism and is known for its sedative and appetite-stimulating properties [15,21,23].

Weight gain associated with antidepressant use is an important clinical consideration, as it can negatively impact treatment adherence and overall health outcomes [24,25]. Patients who experience weight gain may discontinue or avoid treatment, increasing the risk of relapse or worsening depressive symptoms [26,27]. Moreover, antidepressant-induced weight gain can exacerbate comorbid conditions such as obesity, diabetes, and cardiovascular disease [28,29]. Obesity is two to three times more prevalent in individuals with psychiatric disorders, and long-term antidepressant use has been linked to an increased risk of metabolic disorders [29,30]. Given that obesity is the second most common cause of preventable death after smoking, the need to monitor and mitigate antidepressant-induced weight gain is vital. Strategies such as lifestyle interventions and adjunct medications may provide ways that clinicians can manage this adverse effect.

The relationship between antidepressant use and weight gain is complex, as underlying psychiatric conditions such as depression could involve changes in appetite, physical activity, and energy metabolism. However, research suggests that antidepressants can contribute to weight gain through central mechanisms involving serotonergic and dopaminergic pathways [31].

In this article, a critical evaluation is presented on the mechanisms underlying antidepressant-induced weight gain. The major focus is on serotonergic and dopaminergic pathways, comparing weight gain across different classes of antidepressants, including SSRIs, SNRIs, TCAs, MAOIs, and atypical antidepressants. Furthermore, specific risk factors are identified for antidepressant-induced weight gain while evaluating the current available and emerging strategies for weight gain mitigation. The information presented in this article is a comprehensive overview on this subject to inform healthcare providers in their clinical decision-making and will guide future research aimed at balancing antidepressant therapy with metabolic health considerations.

## 2. Methods

A comprehensive literature review was conducted to evaluate the relationship between antidepressant use and weight gain, with a focus on serotonergic and dopaminergic mechanisms, comparative weight effects across medication classes, and strategies for mitigation. The databases PubMed and Google Scholar were utilized to identify relevant studies published primarily between 2000 and 2025, with foundational literature from earlier years included as needed. The following search terms were used alone and in combination: “antidepressant weight gain,” “serotonin and appetite regulation,” “dopamine and feeding behavior,” “SSRIs and metabolism,” “SNRIs and weight gain,” “tricyclic antidepressants and weight gain,” “monoamine oxidase inhibitors and weight change,” “mirtazapine weight gain,” “bupropion weight change,” “antidepressants insulin resistance,” “metabolic effects of antidepressants,” “pharmacogenomics antidepressants,” “gut microbiota and antidepressants,” “GLP-1 receptor agonists antidepressant weight gain,” “adjunctive therapy weight management,” and “lifestyle interventions antidepressants.”

Studies were filtered based on relevance to the review topic. Exclusion criteria included editorials, opinion pieces, and studies lacking detailed methodology or direct reference to weight-related outcomes of antidepressant therapy. Emphasis was placed on high-quality evidence, including randomized controlled trials, meta-analyses, large cohort studies, and systematic reviews.

## 3. Mechanisms Underlying Antidepressant-Induced Weight Gain

### 3.1 Serotonergic Pathways

#### 3.1.1 Role of serotonin in appetite regulation and satiety

Serotonin (5-hydroxytryptamine [5-HT]) has a range of effects in central and peripheral nervous systems [31,32]. Interestingly, the highest concentration of serotonin is found in the GI tract’s enterochromaffin cells [32,33]. Here, serotonin functions to increase intestinal mobilization through stimulation of myenteric neurons, enhancing digestive processes while also reducing appetite [32,34]. Once released into portal circulation, serotonin is taken up into platelets and metabolized by the liver [35].

In the CNS, serotonin plays a role in mood, sleep, and appetite [31]. Beyond appetite regulation, serotonin in the CNS has a significant impact on mood. Its absence is associated with depression, anxiety, and manic episodes [34–36]. It is synthesized from the amino acid tryptophan, which is first converted into 5-hydroxytryptophan (5-HTP) by tryptophan hydroxylase (TPH) and then into serotonin via aromatic acid decarboxylase [37]. These reactions occur in the raphe nuclei of the brainstem, where serotonin is also released. The dorsal raphe nucleus (DRN) contains roughly 35% of serotonergic neurons in the CNS, while the median raphe nucleus (MRN), which contains approximately 8%, and several studies have demonstrated that GABA-A agonism in the MRN increases food intake [38,39].

Serotonin from the rostral nuclei of the serotonergic system regulates various functions, such as temperature, appetite, sleep cycles, emesis, and sexual behavior [36]. These functions are mediated by different serotonin receptors. There are seven serotonin receptor families, all of which act through G-coupled protein receptors except for 5-HT3, which is a ligand-gated ion channel [40]. Serotonin is a pan-agonist to these receptors and influences multiple CNS processes, including appetite regulation [40–42].

The DRN serotonergic neurons suppress appetite through multiple mechanisms, including innervation of the mediobasal hypothalamus [43]. Additionally, the DRN can decrease appetite via projections to the lateral hypothalamic area (LHA) and the bed nucleus of the stria terminalis (BNST) [44]. Serotonergic neurons also stimulate GABAergic neurons in the rostral zona incerta and paraventricular thalamus to inhibit appetite [45]. DRN serotonergic neurons maintain reciprocal connections with other brain regions, including the paraventricular nucleus of the hypothalamus (PVH), lateral hypothalamic area (LHA), arcuate nucleus (ARH), central amygdala (CeA), and parabrachial nucleus (PBN) [46]. A recent study demonstrated that activating the DRN serotonergic pathway to the ARH lead to a decrease of food intake via depolarization of anorexigenic proopiomelanocortin (POMC) neurons while simultaneously hyperpolarizing orexigenic agouti-related peptide (AgRP) neurons [47]. It was found that co-treating mice with fluoxetine and lipocalin 2, an anorexigenic hormone that is used to stimulate melanocortin 4 receptors, led to a normalization of feeding and weight [48].

Different serotonin receptor subtypes have varying effects on food intake. Activation of 5-HT2ARs and 5-HT2CR reduces food intake, whereas 5-HT2BR activation increases it [49–52]. 5-HT2CR agonists stimulate POMC neurons through phospholipase C (PLC) signaling [42,52]. This receptor is also found in the PVH, a region involved in appetite suppression [53,54].

Other, less studied serotonin receptors also influence appetite. 5-HT3R activation increases appetite and food intake in the nucleus accumbens (NAc) but has the opposite effect in the ventral tegmental area (VTA) and nucleus of the solitary tract (NTS) [55,56]. Activation of 5-HT4Rs in the NAc decreases food intake through increased expression of CART mRNA [57]. On the other hand, 5-HT6 increases food intake in the NAc, while 5-HT7 has the opposite effect [58,59].

#### 3.1.2 Alterations due to SSRIs and other serotonergic agents

SSRIs are commonly associated with weight gain. They increase serotonin levels in the synaptic cleft by reducing its reuptake via serotonin transporters in the presynaptic neuron [60,61]. Given serotonin’s role in the CNS, it would be expected that SSRI use would lead to decreased appetite and food intake [62,63]. This is supported by studies in mice injected with fluoxetine, as well as observations in humans during the first few months of SSRI treatment, where some individuals experience weight loss [64–66].

However, more recent studies have shown that chronic SSRI use (≥1 year) is associated with weight gain [67–72]. This adverse effect can impair treatment adherence and have negative implications for overall health [69,70,72].

Several mechanisms have been proposed to explain SSRI-induced weight gain. One mechanism involves serotonin receptor modulation. Long-term fluoxetine (Prozac) use has been shown to downregulate brainstem serotonergic neurons through autoinhibitory signaling via 5-HT1RA. Over extended treatment periods, there is also a decrease in 5-HT2 receptor expression and activity, leading to reduced phosphorylation of CREB and STAT3, along with decreased POMC expression in hypothalamic neurons. This ultimately results in increased food intake and body weight [48]. Other SSRIs, such as paroxetine and citalopram, have been shown to induce weight gain via a similar mechanism [73–75].

Additionally, 5-HT2 receptor desensitization may contribute to weight gain. Studies have shown that pretreatment with 5-HT2 antagonists prevents the anorexic effects of d-fenfluramine (DFF), suggesting that SSRIs that desensitize or downregulate 5-HT2C over time could contribute to increased appetite and weight gain [76]. Supporting this, 5-HT2A receptor agonism promotes satiety, while inhibition leads to increased food intake and weight gain. Furthermore, 5-HT2C receptor antagonism has been associated with glucose intolerance [77], indicating that long-term SSRI or TCA use, which also affects these receptors, may contribute to insulin resistance and weight gain. Long-term SSRI use has also been linked to weight gain due to an increase in carbohydrate cravings [69,78].

**Figure 1:**
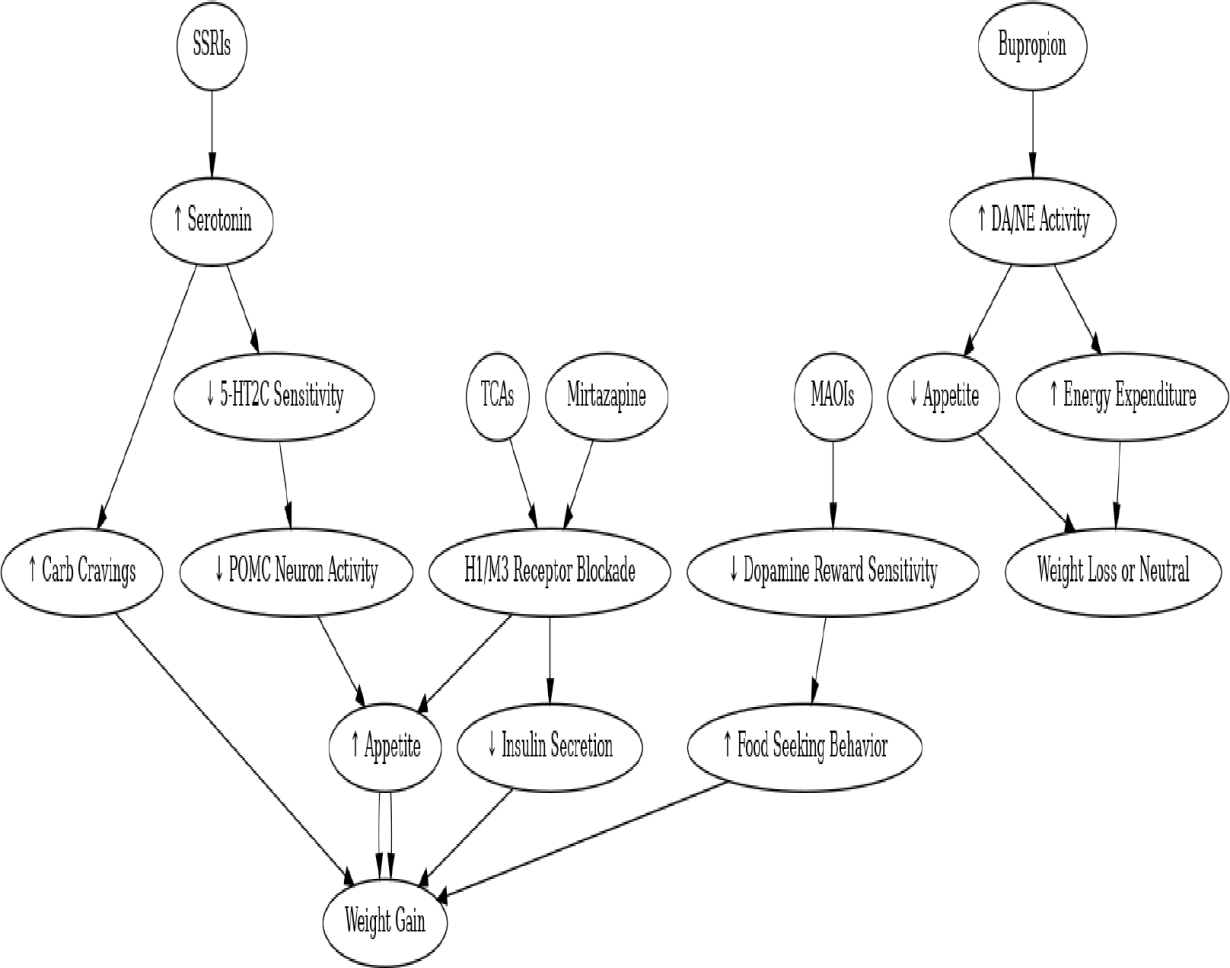
Neurochemical Pathways Linking Antidepressants to Weight Gain.

Another proposed mechanism involves the inhibition of dopamine pathways in the striatum, which can lead to reduced energy expenditure and weight gain [79,80]. Additionally, activation of H1 histamine receptors via citalopram has been correlated with increased food intake [74,75] (Figure 1).

### 3.2 Dopaminergic Pathway

#### Influence on reward circuitry and food-seeking behavior

Food acquisition requires the recognition of rewarding stimuli, and in addition to the serotonergic pathway, the dopaminergic pathways have also been implicated in these processes [81]. Dopamine plays a key role in the reward aspects of feeding through dopaminergic projections from the ventral tegmental area (VTA) to the NAc. Alterations in this system can contribute to weight changes [82,83].

The dopaminergic mesolimbic pathways are essential for feeding behaviors, and disruptions in these pathways can lead to changes in food intake [81]. Dopamine release in the NAc has been observed during feeding, food anticipation, and in response to food-related stimuli [84–86]. Additionally, certain metabolic hormones, such as ghrelin and leptin, can act directly on the VTA to influence feeding behavior. Leptin administration in the VTA has been found to inhibit dopamine activity, leading to a decrease in food intake [87]. In contrast, ghrelin, which is secreted from the stomach and has receptors in the mesolimbic circuits, has the opposite effect—it enhances dopamine release and activity in the VTA, promoting food intake [88]. Furthermore, SSRIs were found to decrease ghrelin levels and alter GI motor activities through 5-HTCR2 receptors which can then lead to changes in feeding behaviors and weight gain [80].

TCAs, which primarily act on dopamine and histamine receptors, have also been associated with weight gain [80,89]. TCAs inhibit H1 histamine and muscarinic acetylcholine receptors, both of which have been linked to increased food intake and weight gain [90].

### 3.3 Hormonal and Metabolic Changes

#### Insulin sensitivity and glucose metabolism involving Leptin and ghrelin dysregulation

Psychotropic medications are associated not only with weight gain but also with metabolic changes such as diabetes and dyslipidemia [91]. Antidepressant use has been linked to changes in insulin resistance, with various antidepressants, including SSRIsm TCAs, and mirtazapine, implicated in the development of these metabolic disorders [91]. Many commonly used antidepressants can lead to insulin resistance (IR) in individuals with and without type 2 diabetes mellitus (T2DM) [92,93]. Specifically, treatment with SSRIs, TCAs, and mirtazapine has been shown to increase cortisol levels, which is associated with increased insulin resistance [94].

Long-term antidepressant use has correlated with an increased risk of T2DM. A case-control study in the United States found that antidepressant use greater than 24 months at moderate-to-high daily doses was associated with an increased risk of developing T2DM compared to non-users [72]. Similarly, a French cohort study with a six-year follow-up found a comparable increased risk of T2DM among antidepressant users, with no significant differences between SSRIs, TCAs, and mixed antidepressants [95].

A 2023 study analyzing the response to vortioxetine, an SSRI, found a significant increase in insulin resistance following treatment [96]. This study also indicated that increased IR contributed to treatment nonresponse and elevated C-reactive protein (CRP) levels, underscoring the metabolic effects of antidepressant therapy. Prior research has shown that CRP may serve as a predictor of antidepressant response [96,97].

**Figure 2:**
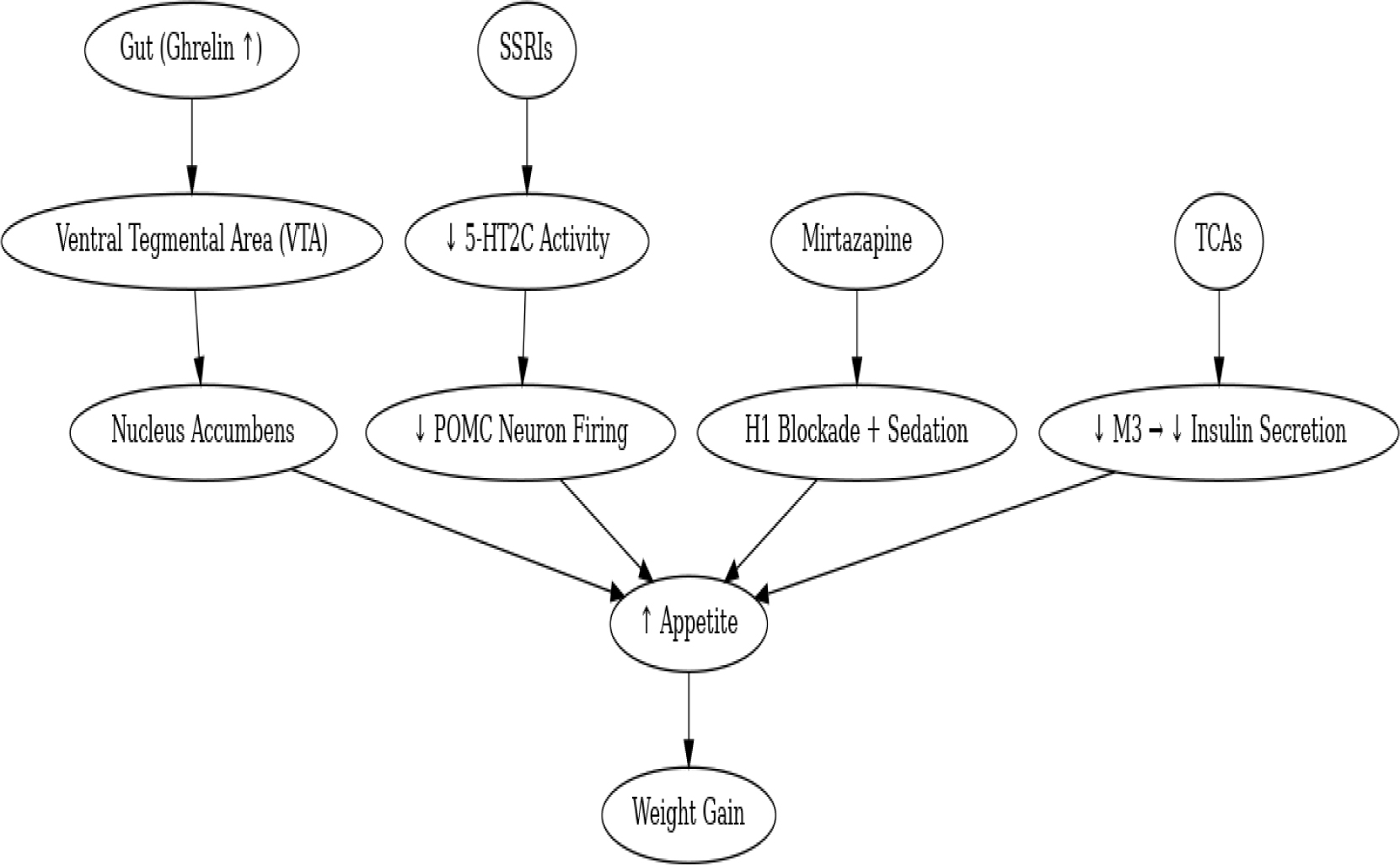
Neurohormonal Interaction Pathways.

TCAs may also influence insulin secretion through their inhibitory effects on M3 muscarinic receptors, which play a key role in insulin release [98]. Notably, antipsychotics that antagonize M3 receptors, such as clozapine, have been associated with the development of T2DM due to decreased insulin secretion [99]. Some TCAs, such as desipramine, have been linked to increased insulin resistance and hyperglycemia [91]. However, interestingly, in a study examining individuals with depression treated with TCAs, there was an observed improvement in IR, suggesting varied metabolic effects [100,101] (Figure 2).

## 4. Extent of Weight Gain Across Antidepressant Classes

Antidepressant-induced weight occurs through alterations in neurotransmitters, metabolic regulation, and behavioral changes. Meta-analysis by Alonso-Pedrero et al. [102] of 27 cohort studies with over 450,000 individuals found that the most significant weight gain was associated with TCAs, mirtazapine, and certain SSRIs. This study concluded that many individuals treated with antidepressants were at increased risk of gaining 5% of their baseline body weight, except for those treated with Bupropion [102]. Another study by Petimar et al. [103] included a target trail emulation study using EHR records from over 180,000 patients to compare weight changes across different antidepressant treatments.

### 4.1 Selective Serotonin Reuptake Inhibitors (SSRIs +SNRIs)

SSRIs are the most prescribed antidepressants but the effect they have on weight gain differs depending on the SSRI. Petimar et al. [103] found that escitalopram had the greatest weight gain over a 6-month period of +0.41kg. Compared to other SSRIs such as, paroxetine with +0.37kg, duloxetine +0.34kg, and venlafaxine +0.17kg. A previous research study by Gafoor et al. [104] found that paroxetine use was associated with a 21% increased risk of at least 5% weight over a 10-year period. Some SSRIs, such as fluoxetine had less of an association with weight changes and remained weight neutral [105].

## 4.2 Tricyclic Antidepressants (TCAs)

TCAs are associated with significant weight gain due to their effects on antihistaminergic and anticholinergic pathways. A study by Alonso-Pedrero et al. [102] found that short-term TCA use (4–12 weeks), specifically amitriptyline, mirtazapine, and nortriptyline had weight gains of +1.52 kg, +1.74 kg, and +2kg respectively. This coincides with other studies that have shown that TCA-associated weight gain has been demonstrated to be dose and time-dependent [106,107]. Specifically, amitriptyline use has been correlated to with continuing weight gain over 24 months and overall, a greater weight change compared to SSRI [107]. Due to the extent of short-term and long-term weight gain associated with TCAs, they are typically avoided in those who are overweight.

### 4.3 Monoamine Oxidase Inhibitors (MAOIs)

MAOIs are less commonly prescribed due to drug-drug interactions and dietary restrictions. They are also associated with weight gain, specifically phenelzine. Phenelzine has been associated with increased weight gain when compared to other classes of antidepressants and has been found to have an increase of 2–3kg over a 6-month course of treatment [102]. This significant increase in weight means that in treatment-resistant depression their consideration needs to be carefully evaluated, especially in patients with comorbidities such as obesity or metabolic syndrome [106].

### 4.4 Atypical Antidepressants

Atypical antidepressants have varying effects on body weight. Mirtazapine and Bupropion are on opposing sides of the weight gain spectrum. Mirtazapine is associated with weight gain and has been correlated with an average increase of +1.74kg in the first 12 weeks of treatment [102]. The mechanism of this weight gain is thought to be through antagonism of H1 and 5HTC receptors, which can lead to increased food intake [108]. On the other hand, Bupropion is associated with a net weight loss. Petimar et al. [103] found that when compared to sertraline there was a −0.22kg weight loss over 6 months [27]. Furthermore, those on Bupropion were found to have a 15% decreased risk of gaining 5% of baseline weight when compared to other SSRIs. Weight protective factors could be due to its unique mechanism of norepinephrine-dopamine reuptake inhibitor, which has been proposed to suppress appetite and increase energy expenditure [109] (Table 1).

## 5. Individual Susceptibility Factors: Genetic predisposition, Lifestyle factors, and Baseline BMI

Certain risk factors such as genetic predisposition, lifestyle factors, and baseline BMI have been associated with antidepressant-induced weight gain and they highlight the need for close monitoring and potential early interventions in patients to prevent excessive weight gain.

A genome-wide association study by Sjaarda et al. [110] has identified four novel loci that were associated with weight gain during psychotropic treatment, including antidepressant treatment [110]. The loci were in proximity of genes that are involved with metabolic regulation, including MAN2A1 and SLCO3A1. Additionally, a retrospective cohort study by Ricardo-Silgado et al. [111] investigated the association between CYP metabolizer phenotypes and weight gain in patients prescribed SSRIs such as citalopram, paroxetine, sertraline, or fluoxetine. The study found that CYP2C19 poor/intermediate metabolizers prescribed citalopram gained significantly more weight (2.6% total body weight gain) compared to normal or rapid/ultra-rapid metabolizers (0.4% and −0.1%, respectively) at six months. These findings indicated that there are genetic predispositions that can influence weight changes in those undergoing antidepressant treatment.

In addition to genetic predisposition, BMI is an important predictor of weight gain during antidepressant use. A higher pretreatment BMI has been associated with a greater weight gain in those treated with psychotropic medications [112]. A study that used machine learning approaches confirmed that in addition to baseline BMI, factors such as age and waist circumference were also significant predictors of weight gain [112].

Lifestyle factors can also play a role in antidepressant-induced weight gain. A study by Simon et al. [113] found that certain lifestyle patterns such as emotional eating, cravings for fast food and sweets, and weight cycling were associated with a higher rate of obesity and metabolic syndrome in psychiatric patients [113]. A different study by Solmi et al. [28] highlighted the importance that lifestyle interventions play in mitigating weight gain associated with antidepressant treatment such as regular exercise and a healthy diet.

## 6. Strategies for Mitigating Antidepressant-Induced Weight Gain

### 6.1 Pharmacological Approaches

Pharmacological approaches to mitigating antidepressant-induced weight gain include switching to weight-neutral or antidepressants or using adjunctive medications targeted at weight control. The atypical antidepressant, bupropion, is consistently associated with the least weight gain among antidepressants and may even lead to weight loss.

Adjunctive medications can also be used for weight control. Metformin is the most used pharmacological treatment for preventing drug related weight gain and has been shown to be effective in reducing weight gain associated with antidepressant use [25,114]. Furthermore, glucagon-like-peptide-1 (GLP-1) receptor agonists, such as liraglutide and exenatide, have been shown to be effective in mitigating weight gain [25,115].

A combination therapy of naltrexone/bupropion (NB) as an adjunct to antidepressant therapy in those with obesity or who were overweight has shown that it can be effective in promoting weight loss with a mean adjusted weight of −6.3% compared to 4.3% in those in the placebo group [116].

The American College of Physicians recommends considering the potential for weight gain when initially selecting antidepressants and suggests switching to less weight-inducing options when necessary [117] (Table 2).

### 6.2 Behavioral and Lifestyle Interventions

In addition to dietary and exercising changes, adding cognitive-behavioral therapy (CBT) has been shown to be efficacious in managing both obesity and comorbid depression. Behavioral strategies should include but not limited to self-monitoring, goal setting, and problem solving to address barriers to weight loss [118].

Early weight gain can predict further weight gain, and regular monitoring of metabolic health at baseline and during follow-ups is important to help identify signs of weight gain to allow for interventions [28].

### 6.3 Emerging and Experimental Interventions: Targeting gut microbiota for weight management - Personalized medicine approaches (pharmacogenomics)

Targeting gut microbiota for weight management in patients with antidepressant-induced weight gain has shown some promising results, although the evidence is still emerging.

Minichino et al. [119] conducted a systematic review and meta-analysis that included studies on antidepressants and their effects on gut microbiota. They found significant changes in gut microbiota diversity metrics following treatment with antidepressants. Specifically, they reported a standard mean difference in alpha diversity of 0.12 (95% CI: 0.01–0.23; p = 0.04; I^2^: 14%) and significant changes in beta diversity (F = 15.59; R^2^ = 0.05; p < 0.001). These changes in gut microbiota composition were associated with differences in efficacy and tolerability of antidepressants, suggesting a potential role in managing weight gain [119].

Nikolova et al. [120] conducted a randomized controlled pilot trial where 49 people with major depressive disorder received either a multi-strain probiotic or placebo for 8 weeks. They found a significant increase in gut microbiota richness in the probiotic group (Chao1 bias-corrected, p = 0.04) and observed between-group differences in beta diversity at week 4 (p = 0.04). However, the study did not specifically measure weight changes [120].

A scoping review by Mötteli et al. [121] found that while probiotics alone did not significantly reduce pharmacologically induced weight gain, synbiotics (a combination of probiotics and prebiotics) included two studies that observed less weight gain in individuals receiving synbiotics compared to those who did not [121].

While the modulation of gut microbiota through synbiotics and probiotics shows potential in managing antidepressant-induced weight gain, more studies are needed to establish definitive clinical guidelines.

Pharmacogenomic treatment approaches to combat antidepressant-induced weight gain involve tailoring antidepressant therapy based on individual genetic profiles to minimize adverse effects such as weight gain.

The retrospective cohort study by Ricardo-Silgado et al. [111] mentioned earlier highlighted the association between CYP metabolizer phenotypes and weight gain in patients prescribed SSRIs such as citalopram, this suggests that pharmacogenomic testing for CYP2C19 could help identify patients at higher risk for weight gain with citalopram, allowing for alternative treatment strategies [111].

Additionally, a genome-wide interaction and enrichment analysis on weight gain during citalopram treatment identified molecular pathways, such as axon guidance and developmental biology, associated with weight gain. Variations in genes involved in collagen synthesis, thyroid hormone activity, energy metabolism, and adipocyte differentiation were implicated, suggesting that genetic profiling could predict weight gain risk and guide personalized treatment [122].

The U.S. Department of Veterans Affairs and U.S. Department of Defense Clinical Practice Guideline reviewed pharmacogenomic testing for antidepressant selection. They concluded that while there is interest in pharmacogenomic approaches, the evidence is currently insufficient to make a strong recommendation for its routine use due to mixed outcomes and low-quality evidence [123].

In summary, pharmacogenomic testing, particularly for CYP2C19, shows potential in predicting and managing antidepressant-induced weight gain, but further research is needed to establish its clinical utility comprehensively.

## 7. Conclusion

Antidepressant-induced weight gain is a significant clinical concern that can impact treatment adherence and overall patient well-being. The underlying mechanisms involve complex interactions between serotonergic, dopaminergic, and metabolic pathways, with different antidepressant classes contributing to varying degrees of weight gain. While antidepressant medications such as TCAs, mirtazapine, and MAOIs are more strongly associated with weight gain, bupropion remains a weight-neutral or weight-reducing option. Individual susceptibility factors can influence the extent of weight change.

Mitigation strategies include switching to weight-neutral antidepressants, implementing pharmacological interventions such as metformin and GLP-1 receptor agonists, and integrating behavioral and lifestyle modifications. Emerging research on pharmacogenomics and gut microbiota offers potential avenues for personalized treatment approaches. However, further longitudinal studies are needed to better understand the long-term metabolic consequences of antidepressant therapy to optimize treatment strategies in order to balance efficacy with metabolic health. Incorporating these insights into clinical practice would enable providers to make informed decisions in order to minimize the burden of weight gain from antidepressant treatment while also effectively managing depression and anxiety disorders.

## Figures and Tables

**Table 1:** Effect of Antidepressants on Weight Change and the potential underlying mechanisms.

Class	Antidepressant	Weight Change (kg)	Risk of a 5% Weight Gain	Mechanism Contributing to Weight Change	Reference
SSRI	Paroxetine	+0.37 to +2.73	21% higher	Strong antihistaminergic effects, appetite stimulation	Petimar et al. [103]; Gafoor et al. [104]
SSRI	Fluoxetine	0.07 to neutral	Neutral	Initial weight loss, potential stabilization	Petimar et al. [103]; Serretti and Mandelli [105]
TCA	Amitriptyline	+1.52 to +2	High	Antihistaminergic and anticholinergic properties	Alonso-Pedrero et al. [102]
TCA	Nortriptyline	+1.52 to +2	High	Antihistaminergic and anticholinergic properties	Alonso-Pedrero et al. [102]
SNRI	Duloxetine	+0.34	10–15% higher risk	Noradrenaline reuptake inhibition, possible metabolic impact	Petimar et al. [103]
SNRI	Venlafaxine	+0.17	Moderate risk	Noradrenergic activity, moderate weight impact	Petimar et al. [103]
MAOI	Phenelzine	+2 to +3	High	Metabolic changes, appetite regulation	Alonso-Pedrero et al. [102]
Atypical	Mirtazapine	+1.74	High	Histamine (H1) and serotonin (5-HT2C) antagonism	Alonso-Pedrero et al. [102]
Atypical	Bupropion	−0.22 to −3.2	15% reduced	Dopamine and norepinephrine reuptake inhibition (appetite suppression)	Petimar et al. 2024 [103]; Aronne et al. [109]

**Table 2:** Pharmacological Mitigation Strategies.

Intervention	Mechanism of Action	Evidence Strength	Common Side Effects	Remarks
Metformin	Insulin sensitizer	Moderate	GI upset	Good for insulin resistance
GLP-1 RAs	Appetite suppression	High	Nausea	Expensive, injectable
Bupropion	DA/NE reuptake inhibition	High	Insomnia	Useful for weight loss
Naltrexone/ Bupropion	Reward pathway modulation	Moderate	Nausea, headache	Combination product

## 8. Key Points

- Long-term antidepressant use is associated with clinically significant weight gain, impacting adherence and long-term health outcomes.
- SSRIs are generally weight-neutral or cause mild weight loss in the short term but are linked to weight gain with prolonged use.
- TCAs, MAOIs, and mirtazapine show the highest propensity for weight gain due to their effects on serotonergic, histaminergic, and dopaminergic systems.
- Bupropion is consistently associated with weight neutrality or modest weight loss, making it a favorable option for patients concerned about weight gain.
- Mechanisms of antidepressant-induced weight gain include 5-HT2 receptor desensitization, dopamine inhibition in reward pathways, increased carbohydrate craving, and hormonal dysregulation (leptin, ghrelin, insulin).
- Genetic predispositions, including CYP2C19 metabolizer status, play a significant role in individual susceptibility to weight changes.
- Higher baseline BMI, emotional eating, and sedentary lifestyle behaviors increase the risk of antidepressant-induced weight gain.
- Metformin and GLP-1 receptor agonists have shown promise as adjunctive pharmacologic strategies to mitigate weight gain.
- Lifestyle interventions such as regular physical activity, dietary modifications, and cognitive-behavioral therapy (CBT) are essential components of management.
- Emerging approaches, including gut microbiota modulation and pharmacogenomic-guided therapy, offer future directions for personalized treatment plans.
